# Bistability of seizure-like bursting and silence

**DOI:** 10.1186/1471-2202-13-S1-P157

**Published:** 2012-07-16

**Authors:** William Barnett, Gabrielle O’Brien, Gennady S Cymbalyuk

**Affiliations:** 1 Neuroscience Institute, Georgia State University, Atlanta, GA, 30303, USA; 2 Agnes Scott College, Decatur, GA, 30030, USA

## 

Heart interneurons (HNs) in the third and fourth ganglia of the medicinal leech are units of the central pattern generator driving the heartbeat. When placed in bath with Co^2+^ and 4-aminopyridine, cells in a ganglion exhibit seizure-like activity with slow plateau oscillations [1]. In this scenario, the synaptic currents, the Ca^2+^ currents, and most of the K^+^ currents are blocked. We consider this bursting activity to be a model for chemically induced seizures.

We present a novel Hodgkin-Huxley type model of the pharmacologically reduced HN. The model contains a leak current and four voltage-gated ionic currents: a fast Na^+^ current, a persistent Na^+^ current (I_P_), a non-inactivating K^+^ current (I_K2_), and a hyperpolarization-activated current (I_h_). This model produced slow plateau-like bursting at an initial set of parameters. We systematically varied two parameters of the leak current (g_Leak_ and E_Leak_) and recorded a variety of silent and oscillatory regimes.

For a given value of E_Leak_, we established the range of values of g_Leak_ for which the system supported bursting activity. For large values of g_Leak_, the model exhibited quiescence. As g_Leak_ was decreased, the equilibrium lost stability in an Andronov-Hopf bifurcation, and a saddle orbit was born. The ranges of values in g_Leak_ for which the system exhibited bursting and quiescence overlapped defining a range of values in g_Leak_ supporting coexistence of the two regimes. Coexistence means that an external perturbation can switch the activity from one of the stable regimes to another.

We explored bistability of bursting and silence by injecting a square pulse of current to perturb bursting activity. Pulses of current were characterized by two parameters: the amplitude of the pulse and the phase in the cycle period at which the pulse was applied. We varied the phase and amplitude through a range of values and determined whether a given pulse switched the activity from bursting to silence. We found contiguous parameter sets that satisfied this criterion.

We examined the role of each current in supporting bistability of bursting and silence. The propensity of the model to bistability of bursting and silence was defined as the range of g_Leak_ for which bursting and silence coexisted. We computed this value while iteratively varying the maximal conductance of each voltage-gated current. Increasing the maximal conductance of I_K2_ increased the range of bistability, and there was a similar trend for I_h_. In contrast, the range of bistability increased as the maximal conductance of I_P_ decreased. We tested whether these effects were complimentary. We selected values of the maximal conductances of I_K2_, I_h_, and I_P_ that each supported the largest range of bistability found. We performed a two-parameter (g_Leak_, E_Leak_) bifurcation analysis in both the initial model and this new model. The range of bistability in the new model increased substantially over that exhibited in the initial model.

We suggest that this study could lead to the development of novel methods for controlling seizures.
